# Supplementary material for: Emergence of decadal linkage between Western Australian coast and Western–central tropical Pacific
Source: Nat Commun. 2024 May 25;15:4458. doi: 10.1038/s41467-024-48900-2 (PMC11128013; doi:10.1038/s41467-024-48900-2)
Supplement: Supplementary file 1 — Supplementary Information [file 41467_2024_48900_MOESM1_ESM.pdf]

Supplementary Information for

Emergence of Decadal Linkage between Western Australian Coast and Western–Central Tropical Pacific

Yuewen Ding, Pengfei Lin, Hailong Liu, Bo Wu, Yuanlong Li, Lin Chen, Lei Zhang, Aixue Hu, Yiming Wang, Yiyun Yao, Bowen Zhao, Wenrong Bai, Weiqing Han

*Corresponding author E-mail: linpf@mail.iap.ac.cn

This PDF file includes the following:

Figs. S1 to S16

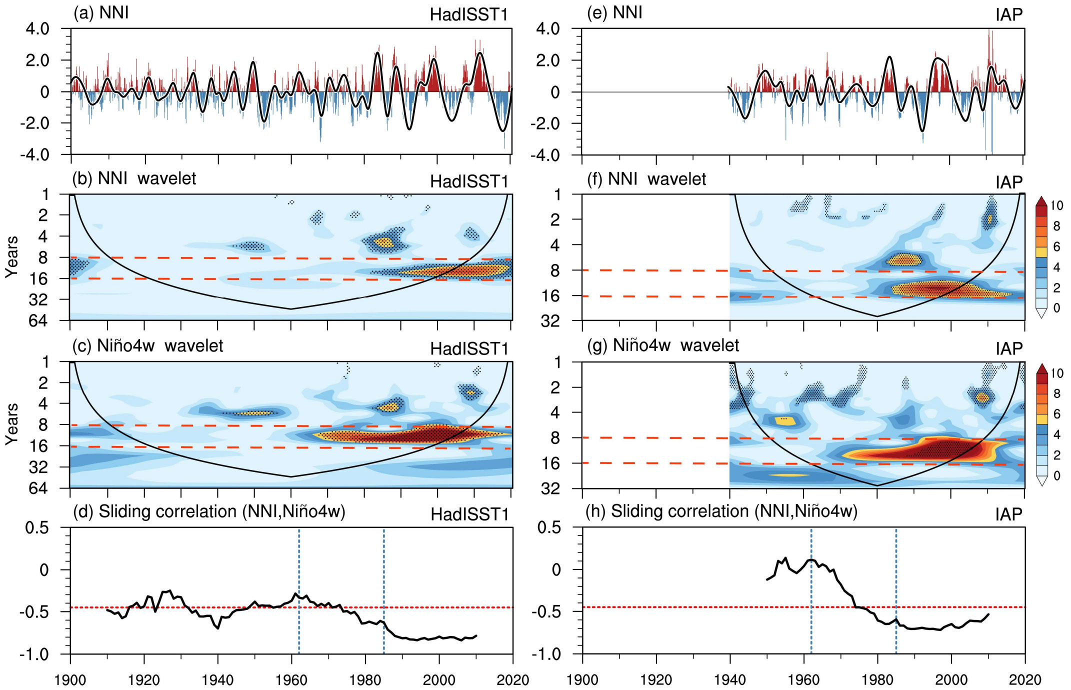

Fig. S1. Sea Surface Temperature (SST) variability off western Australian coast and in the Niño4 west (Niño4w) region, and their relationship in other observed datasets. a Normalized Ningaloo Niño index (NNI; bars) and the decadal component of the NNI from Ensemble empirical mode decomposition (EEMD; black line) in HadISST1. **b** Wavelet spectrum of the NNI in HadISST1. **c** Wavelet spectrum of the Niño4w index in HadISST1. The red lines mark the periodicity of 8–16 years. **d** The 21–year sliding correlation coefficients between the NNI and the Niño4w index in HadISST1. Panels **e–h** are based on IAP data. Source data are provided as a Source Data file.

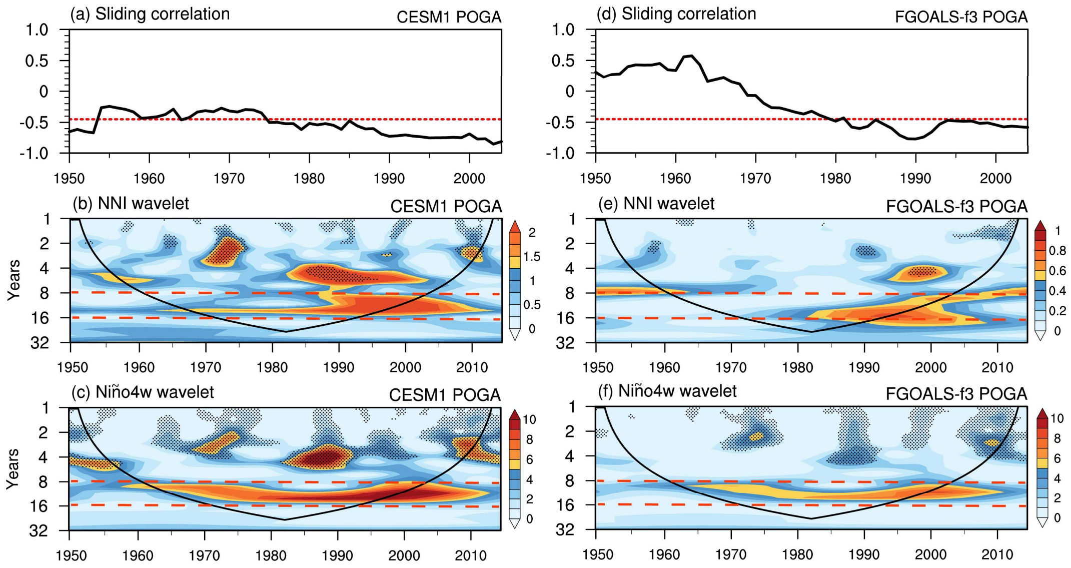

Fig. S2. Sea Surface Temperature (SST) variability off western Australian coast and the western–central tropical Pacific and their relationship in Pacific pacemaker experiments. a The 21-year sliding correlation coefficients between the ensemble mean Ningaloo Niño index (NNI) and the ensemble mean Niño4 west (Niño4w) index in CESM1.2 Pacific Ocean (POGA) pacemaker experiments, in which the red line signifies results statistically significant at the 95% confidence level. **b** Wavelet spectrum of the ensemble mean NNI for 1950–2014 in CESM1.2 Pacific Ocean (POGA) pacemaker experiments. The red lines mark the periodicity of 8–16 years. **c** Wavelet spectrum of the ensemble mean Niño4w index for 1950–2014 in CESM1.2 Pacific Ocean (POGA) pacemaker experiments. **d**, **e**, and **f** as in **a**, **b,** and **c** but in FGOALS-f3-L Pacific Ocean (POGA) pacemaker experiments for 1950–2014. Source data are provided as a Source Data file.

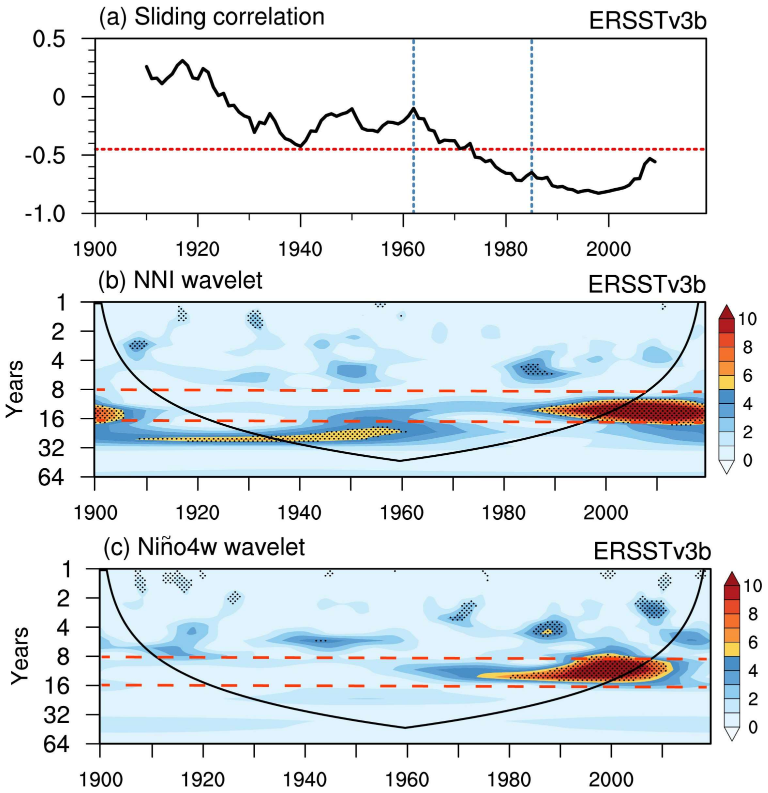

Fig. S3. Sea Surface Temperature (SST) variability off western Australian coast and the tropical western–central tropical Pacific and their relationship in ERSSTv3b. a The 21-year sliding correlation coefficients between the ensemble mean Ningaloo Niño index (NNI) and the ensemble mean Niño4 west (Niño4w) index, in which the red line signifies results statistically significant at the 95% confidence level. **b** Wavelet spectrum of the ensemble mean NNI. The red lines mark the periodicity of 8–16 years. **c** Wavelet spectrum of the ensemble mean Niño4w index. Source data are provided as a Source Data file.

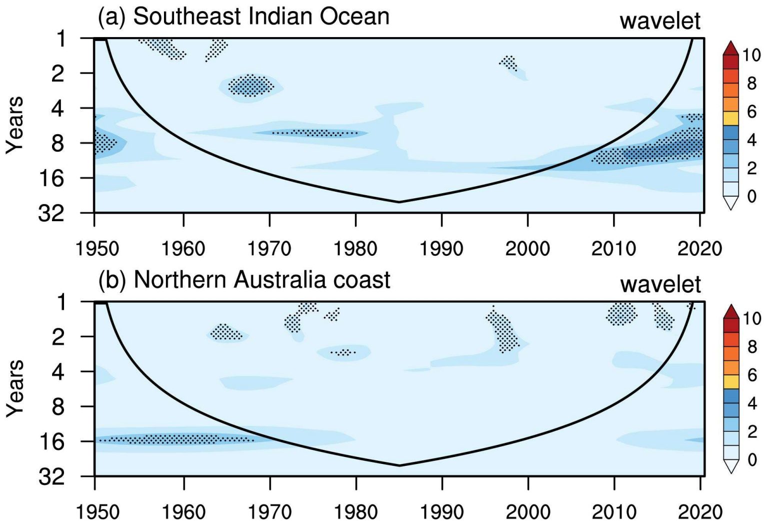

Fig. S4. Sea Surface Temperature (SST) variability in areas around the region off western Australian coast. Wavelet spectrum of **a** southeast Indian Ocean (10°–30°S, 85°–100°E) and **b** northern Australia coast (0°–15°S, 120°–130°E) from 1950–2020 in ERSSTv5. Black dots indicate areas exceeding the 95% confidence intervals. Areas under the cone-shaped lines are influenced by the edge effect. Source data are provided as a Source Data file.

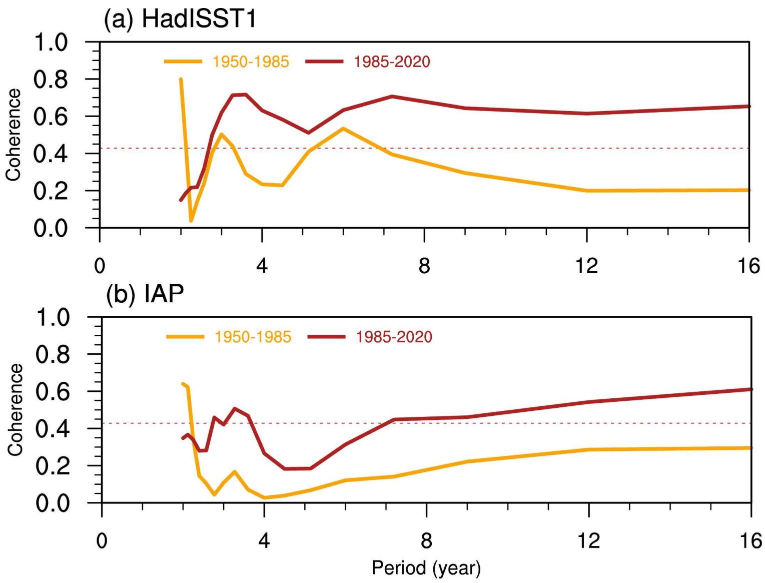

Fig. S5. Covariance between Sea Surface Temperature (SST) variability off western Australian coast and SST variability in the Niño4 west (Niño4w) region. Coherence spectrum of the Ningaloo Niño index (NNI) with the Niño4w index for 1950–1985 and 1985–2020. The red line signifies results statistically significant at the 95% confidence level in **a** HadISST1 **b** IAP data. Source data are provided as a Source Data file.

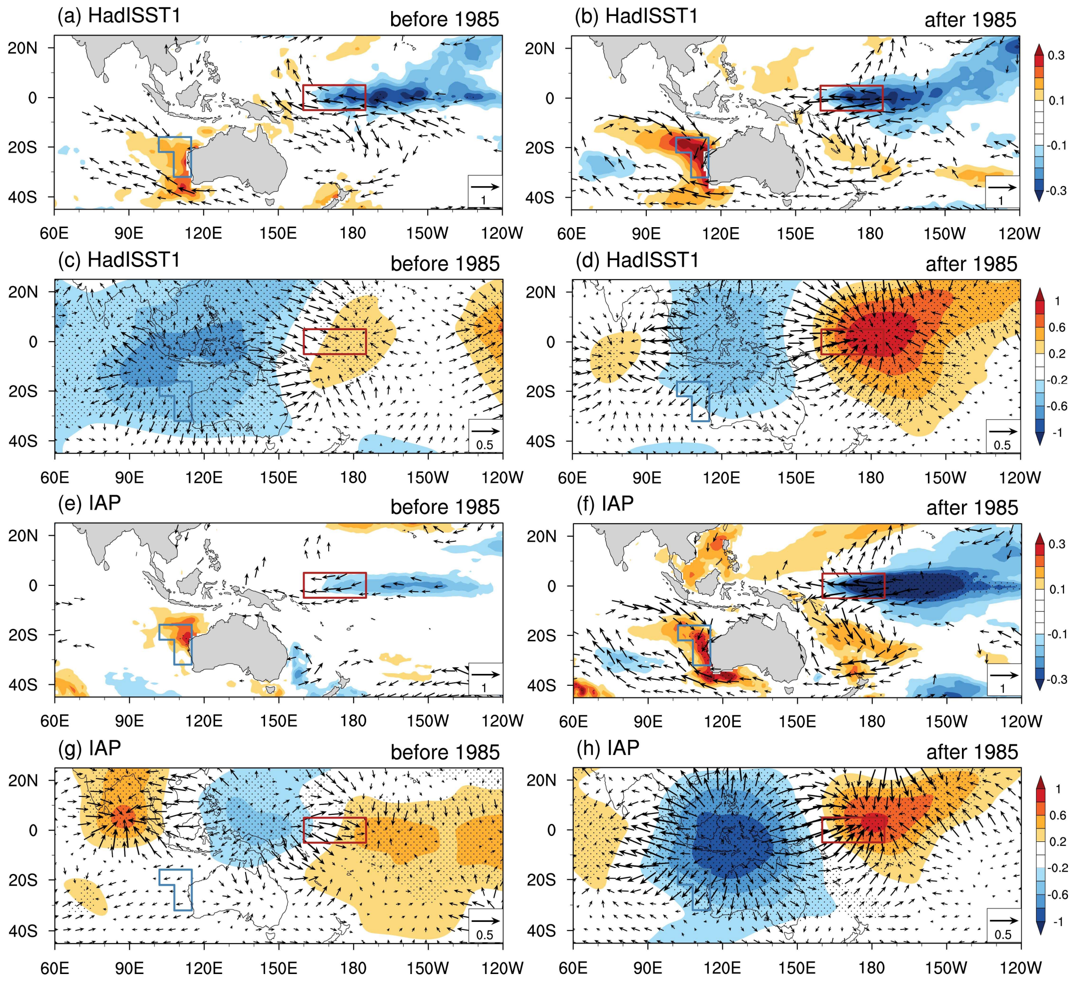

Fig. S6. Teleconnections for decadal Sea Surface Temperature (SST) variability off western Australian coast in December–January–February (DJF) in other observation datasets. Regression of the DJF Sea Surface Temperature Anomaly (SSTA) onto the DJF decadal Ningaloo Niño index (NNI; shading; vectors for wind anomaly) in **a** 1950–1985 and **b** 1985–2020. The blue box represents the region off western Australian coast, and the red box represents the Niño4 west (Niño4w) region. Stippled areas are where the regression results are statistically significant at the 95% confidence level. **c** and **d** as in **a** and **b** but for the 200-hPa velocity potential anomaly (shading; vectors for divergent wind). Panels **a–d** are based on HadISST1, and panels **e–h** are based on IAP data. Source data are provided as a Source Data file.

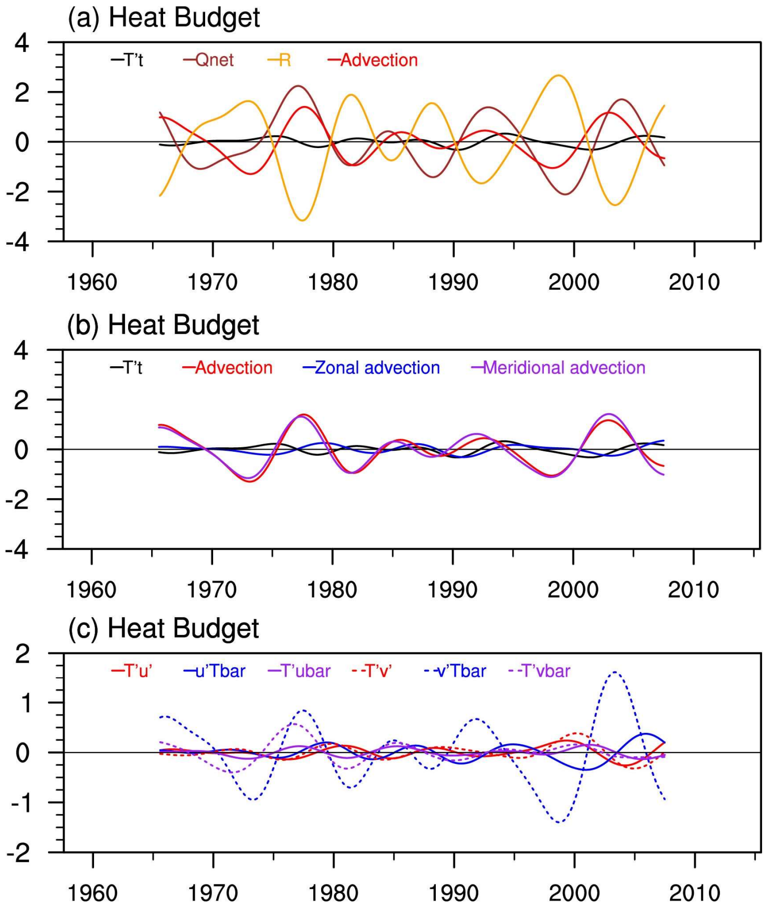

Fig. S7. The contributin of mixed-layer heat budget terms. (a) The timesries *o*f temperature tendency $(\frac{\partial \boldsymbol{T'}}{\partial \boldsymbol{t}})$, net surface heat flux $(\mathbf{Qnet'})$, residual $(\mathbf{R'})$, and the sum of all advection terms. **(b)** The timesries *o*f $\frac{\partial \boldsymbol{T'}}{\partial \boldsymbol{t}}$, the sum of all zonal advection terms, and the sum of all meridional advection terms. (c) individual zonal terms $\boldsymbol{u'}\frac{\partial \boldsymbol{T'}}{\partial \boldsymbol{x}}$, $\boldsymbol{u'}\frac{\partial \overline{\boldsymbol{T}}}{\partial \boldsymbol{x}}$, and $\overline{\boldsymbol{u}}\frac{\partial \boldsymbol{T'}}{\partial \boldsymbol{x}}$; meridional advection terms $\boldsymbol{v'}\frac{\partial \boldsymbol{T'}}{\partial \boldsymbol{y}}$, $\boldsymbol{v'}\frac{\partial \overline{\boldsymbol{T}}}{\partial \boldsymbol{y}}$, and $\overline{\boldsymbol{v}}\frac{\partial \boldsymbol{T'}}{\partial \boldsymbol{y}}$. Monthly data after 8–16 years filtering is used, unit: $\mathbf{10^{-8}}$ K/s. Source data are provided as a Source Data file.

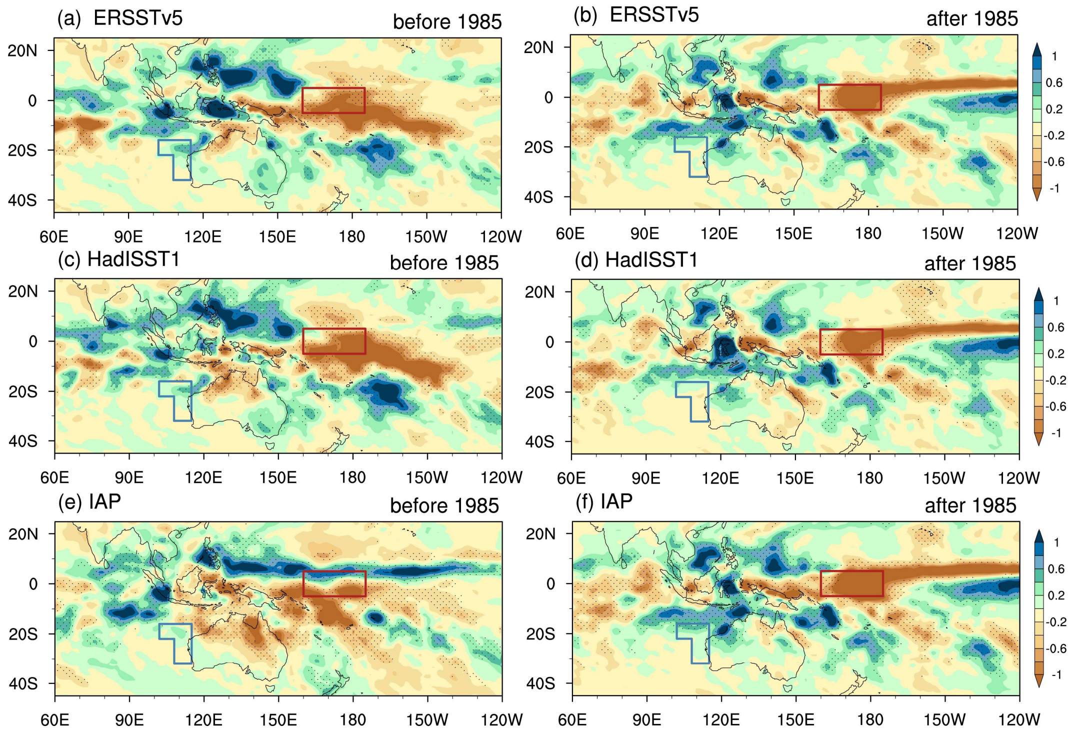

Fig. S8. The increased precipitation over the Maritime Continent. Regression of the December–January–February (DJF) precipitation onto the DJF decadal Ningaloo Niño index (NNI) in **a** 1950–1985 and **b** 1985–2020 for ERSSTv5. Stippled areas are where the regression results are statistically significant at the 95% confidence level. Panels **c** and **d** are based on HadISST1, and panels **e** and **f** are based on IAP data. Source data are provided as a Source Data file.

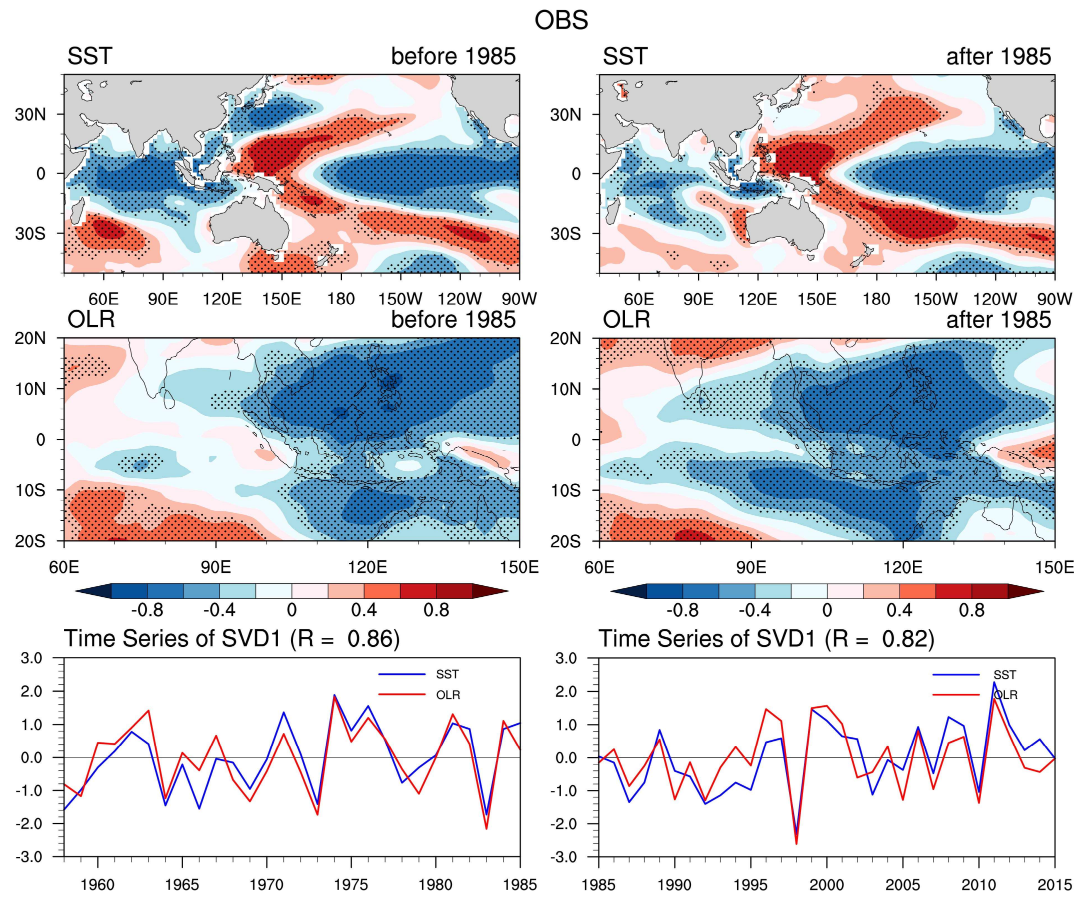

Fig. S9. Connection between Sea Surface Temperature (SST) variability off western Australian coast and convective activity over the Maritime Continent in the observations. The leading singular value decomposition (SVD) modes for the December–January–February (DJF) SST field and the DJF outgoing longwave radiation (OLR) over the Maritime Continent during 1958–1985 (left) and 1985–2015 (right) in observations. Source data are provided as a Source Data file.

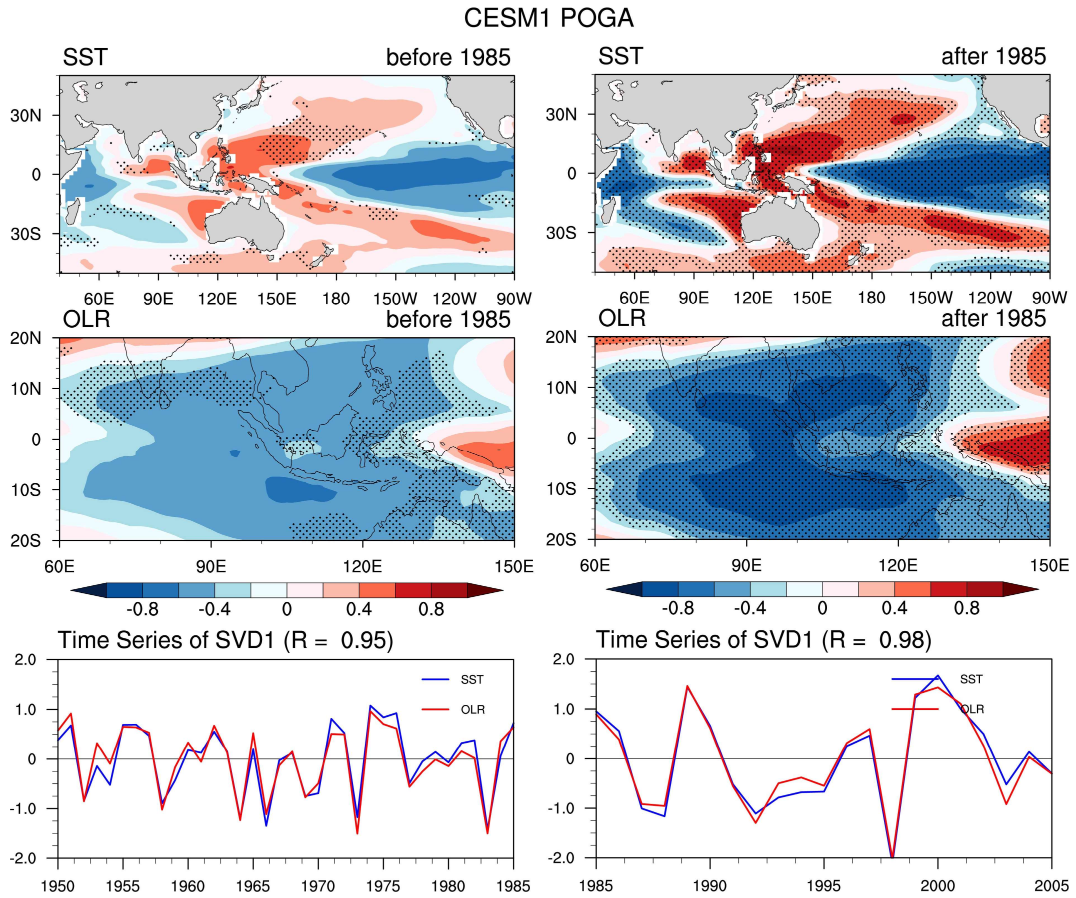

Fig. S10. Connection between Sea Surface Temperature (SST) variability off western Australian coast and convective activity over the Maritime Continent in Pacific pacemaker experiments. a The leading singular value decomposition (SVD) modes for the December–January–February (DJF) SST field and the DJF outgoing longwave radiation (OLR) over the Maritime Continent during 1950–1985 (left) and 1985–2005 (right) in CESM1.2 Pacific Ocean (POGA) pacemaker experiments. Source data are provided as a Source Data file.

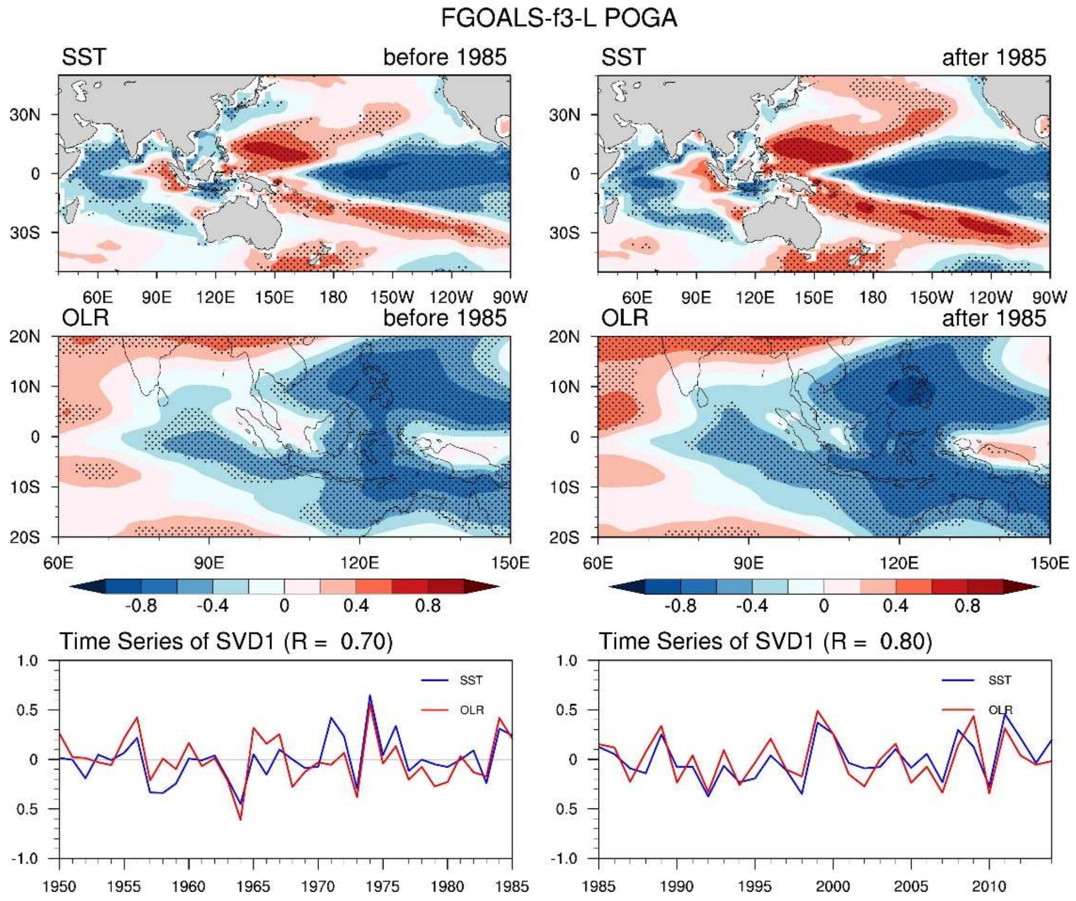

Fig. S11. Connection between SST variability off western Australian coast and convective activity over the Maritime Continent in Pacific pacemaker experiments. a The leading singular value decomposition (SVD) modes for the December–January–February (DJF) SST field and the DJF outgoing longwave radiation (OLR) over the Maritime Continent during 1950–1985 (left) and 1985–2014 (right) in FGOALS-f3-L Pacific Ocean (POGA) pacemaker experiments. Source data are provided as a Source Data file.

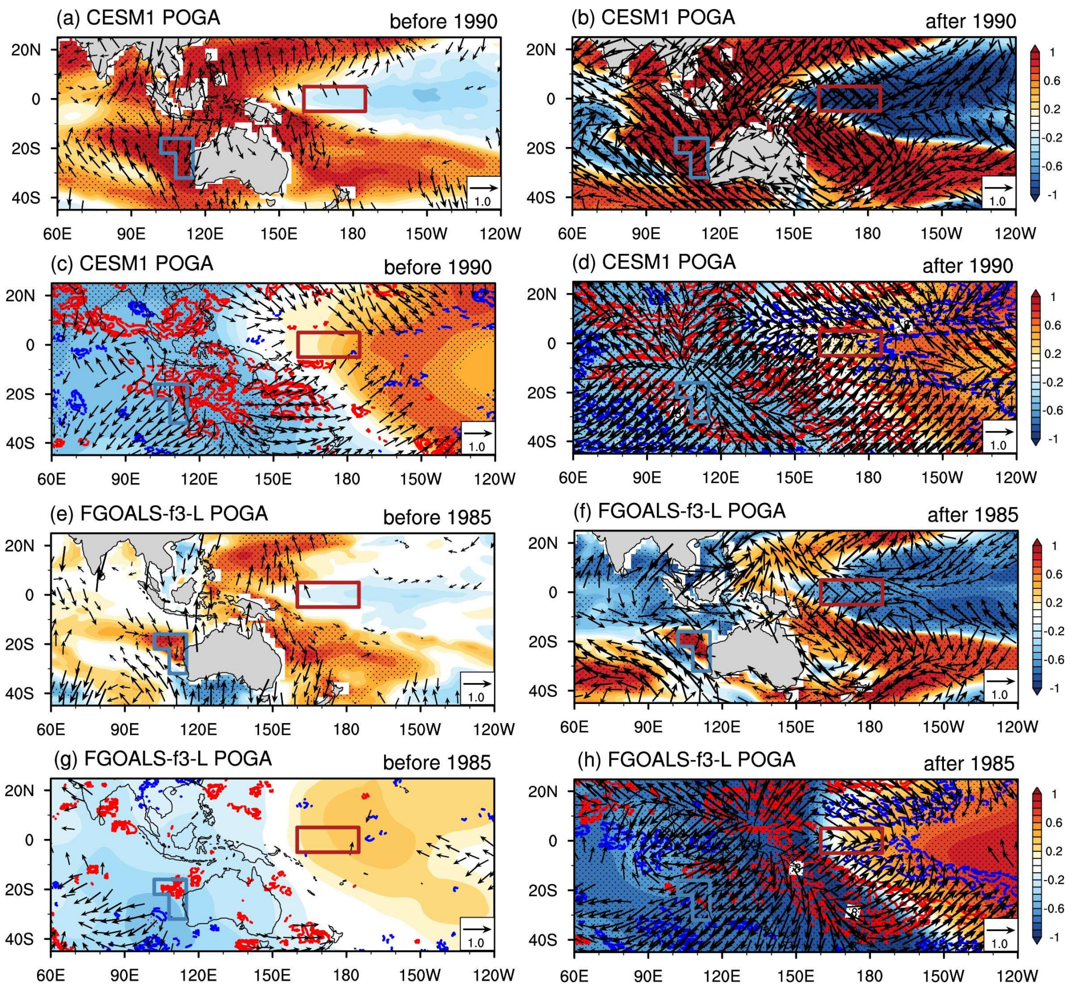

Fig. S12. Teleconnections for decadal Sea Surface Temperature (SST) variability off western Australian coast in December–January–February (DJF) in Pacific pacemaker experiments. Correlation between the DJF decadal Sea Surface Temperature Anomaly (SSTA) and the DJF decadal Ningaloo Niño index (NNI; shading; vectors for wind anomaly; contours for precipitation anomaly) during **a** 1950–1990 and **b** 1990–2014 in CESM1.2 Pacific Ocean (POGA) pacemaker experiments. **c** and **d** as in **a** and **b** but for the 200-hPa velocity potential anomaly (shading; vectors for divergent wind; contours for precipitation). Red/blue contours represent positive/negative precipitation significant at the 95% confidence level. Stippled areas are where the regression results are statistically significant at the 95% confidence level. **e**, **f**, **g**, and **h** are for 1950–1985 and 1985–2014 in FGOALS-f3-L Pacific Ocean (POGA) pacemaker experiments. Source data are provided as a Source Data file.

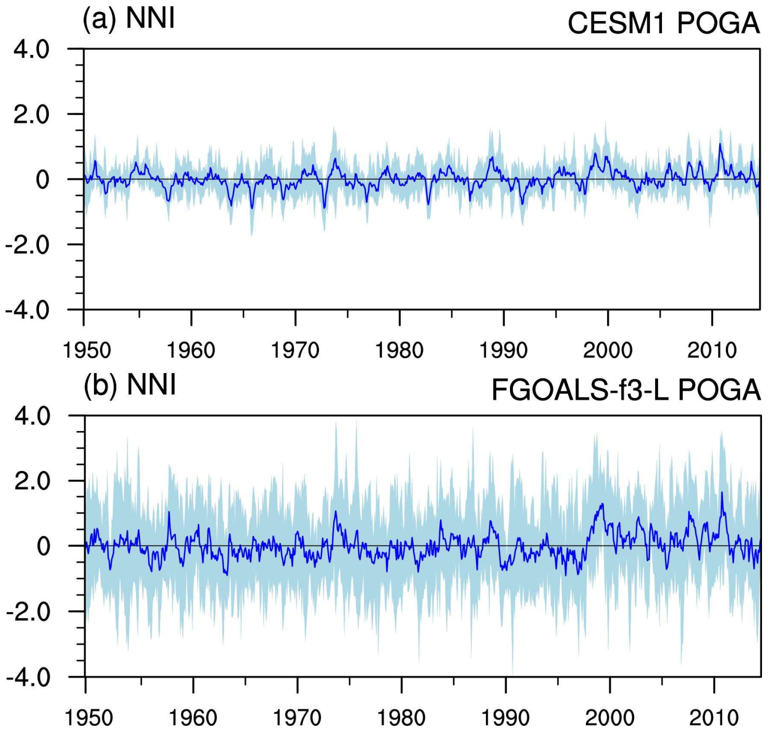

Fig. S13. Variation in the Ningaloo Niño index (NNI) in pacemaker experiments. a The blue line denotes the NNI in CESM1.2 Pacific Ocean (POGA) pacemaker experiments, and the light blue shading denotes the spread of the NNI in CESM1.2 Pacific Ocean (POGA) pacemaker experiments. **b** as in **a** but for FGOALS-f3-L Pacific Ocean (POGA) pacemaker experiments. Source data are provided as a Source Data file.

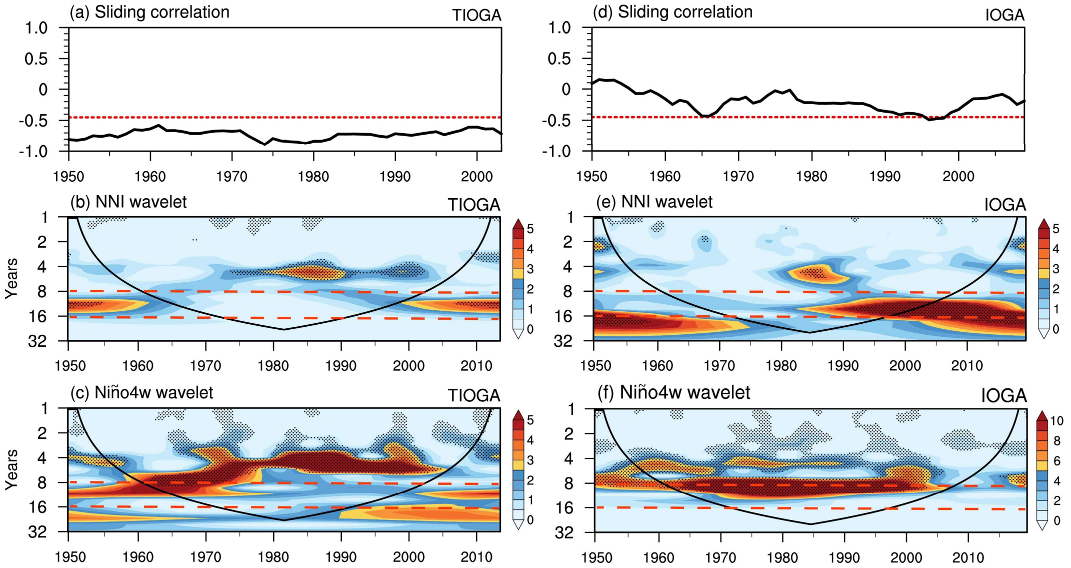

Fig. S14. Sea Surface Temperature (SST) variability off western Australian coast and the western–central tropical Pacific and their relationship in different Indian ocean pacemaker experiments. a The 21-year sliding correlation coefficients between the Ningaloo Niño index (NNI) and the Niño4 west (Niño4w) index in CESM1 tropical Indian ocean (TIOGA) pacemaker experiments, in which the red line signifies results statistically significant at the 95% confidence level. **b** Wavelet spectrum of the NNI for 1950–2013 in TIOGA. The red lines mark the periodicity of 8–16 years. **c** Wavelet spectrum of the Niño4w index for 1950–2013 in TIOGA. **d**, **e**, and **f** as in **a**, **b,** and **c** but in CESM1 Indian ocean (IOGA) pacemaker experiments for 1950–2019. Source data are provided as a Source Data file.

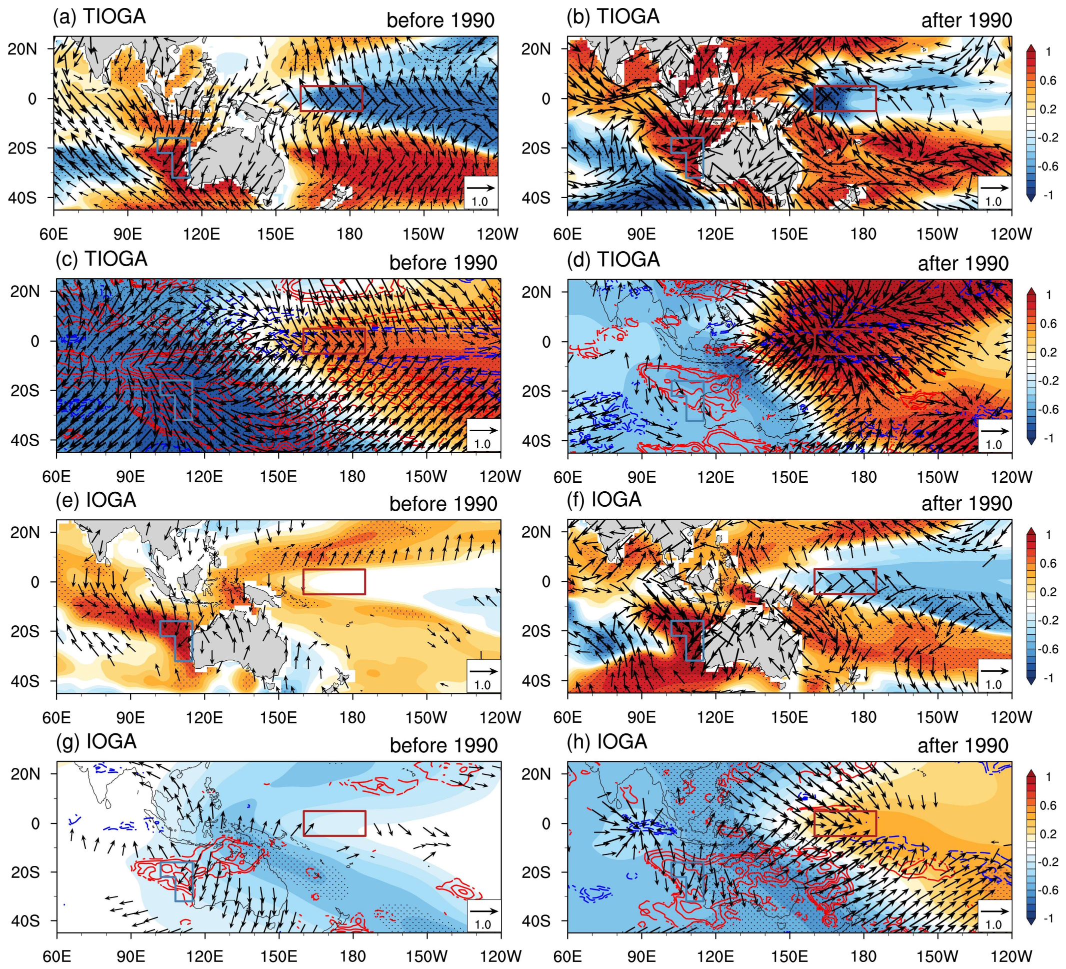

Fig. S15. Teleconnections for decadal Sea Surface Temperature (SST) variability off western Australian coast in December–January–February (DJF) in different Indian Ocean pacemaker experiments. Correlation between the DJF decadal Sea Surface Temperature Anomaly (SSTA) and the DJF decadal Ningaloo Niño index (NNI; shading; vectors for wind anomaly; contours for precipitation anomaly) during **a** 1950–1990 and **b** 1990–2013 in CESM1 tropical Indian ocean (TIOGA) pacemaker experiments. **c** and **d** as in **a** and **b** but for the 200-hPa velocity potential anomaly (shading; vectors for divergent wind; contours for precipitation). Red/blue contours represent positive/negative precipitation significant at the 95% confidence level. Stippled areas are where the regression results are statistically significant at the 95% confidence level. **e**, **f**, **g**, and **h** are for 1950–1990 and 1990–2019 in CESM1 Indian ocean (IOGA) pacemaker experiments. Source data are provided as a Source Data file.

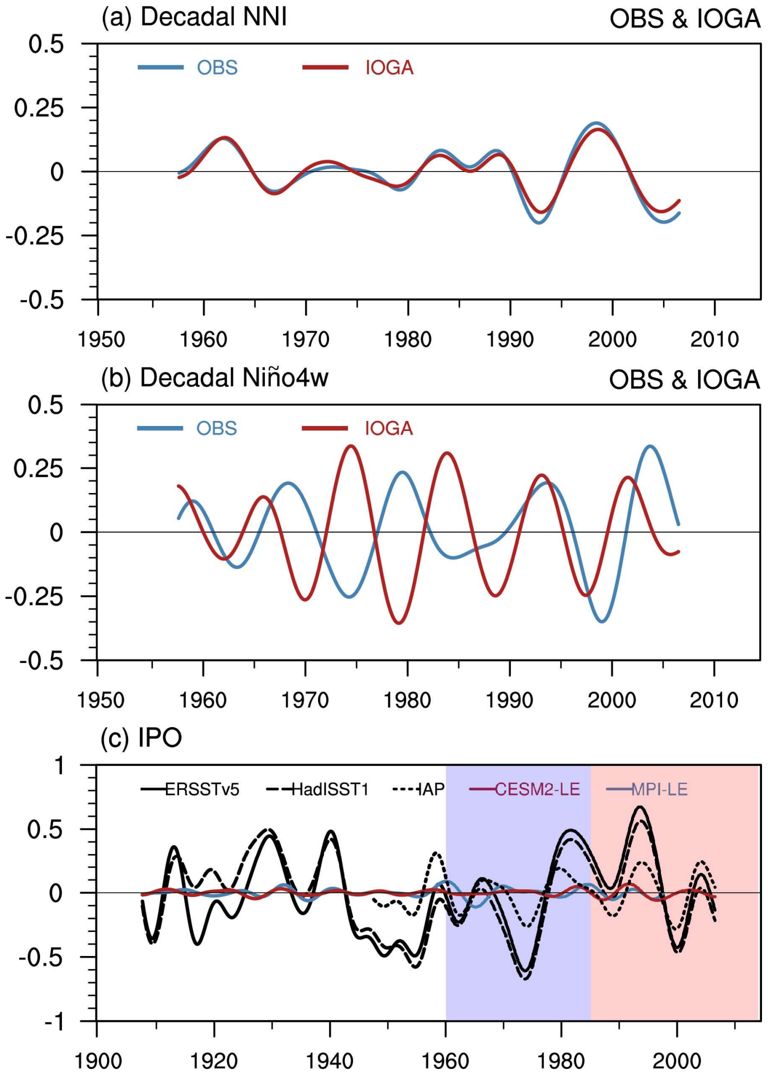

Fig. S16. Decadal Ningaloo Niño index (NNI) and Niño4 west (Niño4w) index in Indian ocean (IOGA) pacemaker experiments and observation; Interdecadal Pacific Oscillation (IPO) indexes in two large ensembles and different observation data. a The 8–16 years band-pass filtered decadal NNI in ERSSTv5 and IOGA. **b** The 8–16 years band-pass filtered decadal Niño4w index in ERSSTv5 and IOGA. **c** The 8 years low-pass filtered IPO index (15°N–15°S, 50°–160°E) in observations and the ensemble mean of CESM2-LE, and MPI-LE. Light blue shading identifies the period 1960-1985. Light red shading identifies the period after 1985. Source data are provided as a Source Data file.
