# Supplementary material for: Emergence of decadal linkage between Western Australian coast and Western–central tropical Pacific
Source: Nat Commun. 2024 May 25;15:4458. doi: 10.1038/s41467-024-48900-2 (PMC11128013; doi:10.1038/s41467-024-48900-2)
Supplement: Supplementary file 3 — Reporting Summary [file 41467_2024_48900_MOESM3_ESM.pdf]

nature portfolio

Corresponding author(s): Pengfei Lin

Last updated by author(s): May 5, 2024

Reporting Summary

Nature Portfolio wishes to improve the reproducibility of the work that we publish. This form provides structure for consistency and transparency in reporting. For further information on Nature Portfolio policies, see our Editorial Policies and the Editorial Policy Checklist.

Statistics

For all statistical analyses, confirm that the following items are present in the figure legend, table legend, main text, or Methods section.

n/a	Confirmed	
[ ]	[x]	The exact sample size (*n*) for each experimental group/condition, given as a discrete number and unit of measurement
[x]	[ ]	A statement on whether measurements were taken from distinct samples or whether the same sample was measured repeatedly
[ ]	[x]	The statistical test(s) used AND whether they are one- or two-sided<br>*Only common tests should be described solely by name; describe more complex techniques in the Methods section.*
[x]	[ ]	A description of all covariates tested
[x]	[ ]	A description of any assumptions or corrections, such as tests of normality and adjustment for multiple comparisons
[ ]	[x]	A full description of the statistical parameters including central tendency (e.g. means) or other basic estimates (e.g. regression coefficient) AND variation (e.g. standard deviation) or associated estimates of uncertainty (e.g. confidence intervals)
[ ]	[x]	For null hypothesis testing, the test statistic (e.g. *F*, *t*, *r*) with confidence intervals, effect sizes, degrees of freedom and *P* value noted<br>*Give P values as exact values whenever suitable.*
[x]	[ ]	For Bayesian analysis, information on the choice of priors and Markov chain Monte Carlo settings
[x]	[ ]	For hierarchical and complex designs, identification of the appropriate level for tests and full reporting of outcomes
[x]	[ ]	Estimates of effect sizes (e.g. Cohen's *d*, Pearson's *r*), indicating how they were calculated

Our web collection on statistics for biologists contains articles on many of the points above.

Software and code

Policy information about availability of computer code

Data collection	All observational and model datasets used in this study are available publicly or on request. Observational data are available from NOAA (https://psl.noaa.gov/data/gridded/data.noaa.ersst.v5.html and https://climatedataguide.ucar.edu/climate-data/sst-data-noaa-extended-reconstruction-ssts-version-3-ersstv3-3b), the Hadley Centre (https://www.metoffice.gov.uk/hadobs/hadisst/), the NCEP/NCAR Reanalysis Project (https://psl.noaa.gov/data/reanalysis/reanalysis.shtml), and the Japan Meteorological Agency (https://jra.kishou.go.jp/JRA-55/index_en.html). The Data from IAP observation is available via http://www.ocean.iap.ac.cn/pages/dataService/dataService.html. The data from ORAs5 is available via https://cds.climate.copernicus.eu/cdsapp#!/dataset/reanalysis-oras5?tab=form. The CESM2 large ensemble datasets are available via https://www.cesm.ucar.edu/projects/community-projects/LENS2/data-sets.html. The MPI large ensemble datasets are available via https://esgf-data.dkrz.de/projects/mpi-ge/. The four single forcing large ensembles are available via https://www.cesm.ucar.edu/working-groups/climate/simulations/cesm2-single-forcing-le. The tropical Indian Ocean pacemaker experiment (TIOGA) datasets are available via https://www.cesm.ucar.edu/working-groups/climate/simulations/cesm1-indian-ocean-pacemaker. The related data of some pacemaker experiments (CESM1 Pacific pacemaker experiment, FGOALS-f3 Pacific pacemaker experiment, and Indian Ocean pacemaker experiment) are available via https://doi.org/10.57760/sciencedb.07476.
Data analysis	Statistics and other calculations were conducted using NCAR Command Language (NCL).

For manuscripts utilizing custom algorithms or software that are central to the research but not yet described in published literature, software must be made available to editors and reviewers. We strongly encourage code deposition in a community repository (e.g. GitHub). See the Nature Portfolio guidelines for submitting code & software for further information.

Data

Policy information about availability of data

All manuscripts must include a data availability statement. This statement should provide the following information, where applicable:

- Accession codes, unique identifiers, or web links for publicly available datasets
- A description of any restrictions on data availability
- For clinical datasets or third party data, please ensure that the statement adheres to our policy

All observational and model datasets used in this study are available publicly or on request. Observational data are available from NOAA (https://psl.noaa.gov/data/gridded/data.noaa.ersst.v5.html and https://climatedataguide.ucar.edu/climate-data/sst-data-noaa-extended-reconstruction-ssts-version-3-ersstv3-3b), the Hadley Centre (https://www.metoffice.gov.uk/hadobs/hadisst/), the NCEP/NCAR Reanalysis Project (https://psl.noaa.gov/data/reanalysis/reanalysis.shtml), and the Japan Meteorological Agency (https://jra.kishou.go.jp/JRA-55/index_en.html). The Data from IAP observation is available via http://www.ocean.iap.ac.cn/pages/dataService/dataService.html. The data from ORAs5 is available via https://cds.climate.copernicus.eu/cdsapp#!/dataset/reanalysis-oras5?tab=form. The CESM2 large ensemble datasets are available via https://www.cesm.ucar.edu/projects/community-projects/LENS2/data-sets.html. The MPI large ensemble datasets are available via https://esgf-data.dkrz.de/projects/mpi-ge/. The four single forcing large ensembles are available via https://www.cesm.ucar.edu/working-groups/climate/simulations/cesm2-single-forcing-le. The tropical Indian Ocean pacemaker experiment (TIOGA) datasets are available via https://www.cesm.ucar.edu/working-groups/climate/simulations/cesm1-indian-ocean-pacemaker. The related data of some pacemaker experiments (CESM1 Pacific pacemaker experiment, FGOALS-f3 Pacific pacemaker experiment, and Indian Ocean pacemaker experiment) are available via https://doi.org/10.57760/sciencedb.07476.

Research involving human participants, their data, or biological material

Policy information about studies with human participants or human data. See also policy information about sex, gender (identity/presentation), and sexual orientation and race, ethnicity and racism.

Reporting on sex and gender	NA
Reporting on race, ethnicity, or other socially relevant groupings	NA
Population characteristics	NA
Recruitment	NA
Ethics oversight	NA

Note that full information on the approval of the study protocol must also be provided in the manuscript.

Field-specific reporting

Please select the one below that is the best fit for your research. If you are not sure, read the appropriate sections before making your selection.

☐ Life sciences ☐ Behavioural & social sciences ☒ Ecological, evolutionary & environmental sciences

For a reference copy of the document with all sections, see nature.com/documents/nr-reporting-summary-flat.pdf

Ecological, evolutionary & environmental sciences study design

All studies must disclose on these points even when the disclosure is negative.

Study description	Community Earth System Model, version 1 (CESM1) from NCAR, and the low-resolution version of the Chinese Academy of Sciences (CAS) Flexible Global Ocean–Atmosphere–Land System model, finite-volume version 3 (FGOALS-f3-L). The model SSTs for the tropical central-eastern Pacific (20°N–20°S, 175°E–75°W) are restored to the model's climatological mean plus the observed anomaly (HadISST1). Tropical Indian Ocean Global-Atmosphere (TIOGA) using CESM1, where SST in the tropical Indian Ocean and part of the western Pacific warm pool region is restored to observed SST from Extended Reconstructed SST version 3b (ERSSTv3b), with fully restored SSTA for 15°N-15°S, from the African Coast to 161°E, and buffer zones at 15°N-20°N, 15°S-20°S, and 161°E-180°. Another experiment is IOGA (full Indian Ocean Global-Atmosphere) using CESM1, where SST in the Indian Ocean (north of 50°S) is restored to observed SSTA from ERSSTv3b. Meanwhile, the oceanic and atmospheric components are freely coupled in other ocean areas.
Research sample	NA
Sampling strategy	NA
Data collection	All observational and model datasets used in this study are available publicly or on request. Observational data are available from NOAA (https://psl.noaa.gov/data/gridded/data.noaa.ersst.v5.html), the Hadley Centre (https://www.metoffice.gov.uk/hadobs/hadisst/), the NCEP/NCAR Reanalysis Project (https://psl.noaa.gov/data/reanalysis/reanalysis.shtml), and the Japan Meteorological Agency (https://jra.kishou.go.jp/JRA-55/index_en.html). The Data from IAP observation is available via http://www.ocean.iap.ac.cn/pages/dataService/dataService.html. The data from ORAs5 is available via https://cds.climate.copernicus.eu/cdsapp#!/dataset/reanalysis-oras5?tab=form. The CESM2 large ensemble datasets are available via https://www.cesm.ucar.edu/projects/community-

	projects/LENS2/data-sets.html. The MPI large ensemble datasets are available via https://esgf-data.dkrz.de/projects/mpi-ge/. The four single forcing large ensembles are available via https://www.cesm.ucar.edu/working-groups/climate/simulations/cesm2-single-forcing-le. The tropical Indian Ocean pacemaker experiment (TIOGA) datasets are available via https://www.cesm.ucar.edu/working-groups/climate/simulations/cesm1-indian-ocean-pacemaker. The related data of some pacemaker experiments (CESM1 Pacific pacemaker experiment, FGOALS-f3 Pacific pacemaker experiment, and Indian Ocean pacemaker experiment) are available via https://doi.org/10.57760/sciencedb.07476.
Timing and spatial scale	Historical radiative forcing is used to drive CESM1 pacemaker (CESM1 POGA, with eight members) for 1870–2005 (is extended to 2014), and FGOALS-f3-L pacemaker (FGOALS-f3 POGA, with ten members) for 1870–2014. TIOGA (ten members) is from 1920 to 2013. IOGA (ten members) is from 1920-2019.
Data exclusions	NA
Reproducibility	The study data is from the numerical simulation experiments. The simulation experiments can be reproduced if the same numerical simulation experiment is done. If the researcher follows the steps to do the numerical simulation experiment using the same climate model, the similar results will be obtained.
Randomization	The numerical simulation experiments have some random noise. This noise can be amplified by non-linear interactions. By using different initial values only in a single model and the same physical processes to do several experiments, the noise can be controlled. In our study, the random is considered in the experiments and controlled by the analysis.
Blinding	Blinding is not applicable to this study as it involves a numerical simulation experiments, which does not include experimental interventions or human subjects.

Did the study involve field work? ☐ Yes ☒ No

Reporting for specific materials, systems and methods

We require information from authors about some types of materials, experimental systems and methods used in many studies. Here, indicate whether each material, system or method listed is relevant to your study. If you are not sure if a list item applies to your research, read the appropriate section before selecting a response.

Materials & experimental systems

n/a	Involved in the study
☒	☐ Antibodies
☒	☐ Eukaryotic cell lines
☒	☐ Palaeontology and archaeology
☒	☐ Animals and other organisms
☒	☐ Clinical data
☒	☐ Dual use research of concern
☒	☐ Plants

Methods

n/a	Involved in the study
☒	☐ ChIP-seq
☒	☐ Flow cytometry
☒	☐ MRI-based neuroimaging

Plants

Seed stocks	NA
Novel plant genotypes	NA
Authentication	NA
